# Frequent co-occurrence of high-grade dysplasia in large flat colonic polyps (>20 mm) and synchronous polyps

**DOI:** 10.1186/s12876-015-0312-4

**Published:** 2015-07-10

**Authors:** Tianzuo Zhan, Felix Hahn, Thomas Hielscher, Johannes Betge, Georg Kähler, Matthias P. Ebert, Sebastian Belle

**Affiliations:** 1 Division of Signaling and Functional Genomics, German Cancer Research Center (DKFZ), Im Neuenheimer Feld 580, D-69120 Heidelberg, Germany; 2 Department of Internal Medicine II, Universitätsmedizin Mannheim, Medical Faculty Mannheim, Heidelberg University, Theodor-Kutzer Ufer 1-3, D-68167 Mannheim, Germany; 3 Division of Biostatistics, German Cancer Research Center (DKFZ), Im Neuenheimer Feld 280, D-69120 Heidelberg, Germany; 4 Central Interdisciplinary Endoscopy Unit, Universitätsmedizin Mannheim, Medical Faculty Mannheim, Heidelberg University, Theodor-Kutzer Ufer 1-3, D-68167 Mannheim, Germany

**Keywords:** Colonic polyp, Colonoscopy, Adenoma, Synchronous polyps, Endoscopic mucosal resection

## Abstract

**Background:**

Large colonic polyps are associated with advanced dysplasia, but prevalence and characteristics of synchronous polyps in patients with large flat colonic polyps are poorly investigated. This study aims to characterize clinicopathological features of large flat colonic polyps and their impact on occurrence and characteristics of synchronous polyps.

**Methods:**

A total of 802 patients that underwent endoscopic mucosal resection (EMR) of flat colonic polyps >20 mm from 2003 to 2014 in an academic endoscopy unit were retrospectively analyzed for size, location and histology of large polyps and synchronous polyps.

**Results:**

Average size of large polyps was 34.1 mm (range 20–150 mm, standard deviation 16.1 mm). Histology included 52.5 % adenomas with low-grade dysplasia (LGD), 26.7 % with high-grade dysplasia (HGD), 9.6 % serrated polyps and 11.2 % adenocarcinomas. The majority of large polyps were localized in the proximal colon (61 %). 72.2 % of adenocarcinomas were found in the distal colon, while 80.5 % of all serrated polyps were detected in the proximal colon. Increase in polyp size, advanced age and location in the distal colon were associated with presence of HGD/adenocarcinoma in large polyps, as identified by multivariate analysis. Synchronous polyps were detected in 67.2 % of patients undergoing complete colonoscopy during EMR. Presence of HGD/adenocarcinoma in the large polyp, localization of any synchronous polyp in the rectosigmoid colon and occurrence of multiple synchronous polyps were associated with presence of HGD/adenocarcinoma in synchronous polyps.

**Conclusions:**

Synchronous polyps are frequently found in patients with large flat colonic polyps. The prevalence of synchronous polyps with high grade dysplasia is highest in patients with large flat polyps containing HGD/adenocarcinoma.

**Electronic supplementary material:**

The online version of this article (doi:10.1186/s12876-015-0312-4) contains supplementary material, which is available to authorized users.

## Background

Despite all activities in prevention, colorectal cancer is still one of the leading causes of cancer-associated morbidity and mortality worldwide [1, 2]. The main route of colorectal cancer development is a progression from adenoma with low to high-grade dysplasia to adenocarcinoma [3]. Depending on the site of occurrence, specific histological subtypes and driver mutations can be found. Adenomatous polyps for instance are more frequent in the distal part of the colorectum and usually have mutations in the APC gene, while serrated polyps are predominantly located in the proximal colon and show a high prevalence of BRAF mutations [4, 5].

Screening colonoscopy has been proven to be effective in reducing death from colorectal cancer by detection and removal of early polyps [6]. Colonic polyps are found in approximately 20–49 % of asymptomatic patients undergoing screening colonoscopy, most of which are adenomas [7–9]. Small lesions <10 mm account for the majority of detected polyps and rarely harbor high-grade dysplasia [7, 10]. The prevalence of advanced dysplasia increases with adenoma size [7, 11]. Several studies show that large flat adenomas >20 mm are associated with a high proportion of high-grade dysplasia and carcinoma in situ [12–14]. However, there is a considerable variance in frequency between studies, ranging from 8 % [15] to over 60 % [14]. In addition, some studies described a preference of large flat adenomas to occur in the right colon [12, 16, 17], while others found a predominantly left sided localization [14, 18]. Finally, the correlation between histological subtypes and localization has been described by a few studies, showing that high grade dysplasia and carcinoma in large flat polyps predominantly occur in the left colon [11, 13, 16].

Synchronous polyps are found in approximately 15–36 % of all screening colonoscopies [19, 20]. In patients with colorectal cancer, occurrence of synchronous polyps correlates with incidence of synchronous and metachronous cancer [19]. Furthermore, the incidence of non-invasive and invasive colorectal carcinoma is higher in patients with multiple synchronous polyps [8]. Thus, the occurrence of synchronous polyps and factors associated with the presence of synchronous polyps are clinically relevant. Recently, several studies showed that the presence of large serrated polyps (>10 mm) in the proximal colon is associated with synchronous polyps containing advanced dysplasia [20–22]. Whether this observation applies on large colonic adenomas in general has so far not been sufficiently investigated.

We therefore aimed to characterize localization and histopathology of flat colonic polyps >20 mm in a large single center cohort. Our goal was to identify associations between histology and location of large flat polyps with prevalence and characteristics of synchronous polyps.

## Methods

### Ethical approval

This study is part of a project investigating life style associated risk factors for large adenomas and has been approved by the local board of ethics (Medizinische Ethikkomission II, Heidelberg University, identifier: 2013–557 N-MA) and is in accordance with the Treaty of Helsinki.

### Data collection

The electronic database of the Central Interdisciplinary Endoscopy Unit of Mannheim University Hospital, Heidelberg University, was reviewed for all patients who underwent endoscopic mucosal resection (EMR) from January 2003 to January 2014. All patients with colonic polyps >20 mm in maximal dimension were included in the preliminary review. Polyp size was determined during endoscopy by comparing polyps with forceps or snares as reference. EMRs of the large colonic polyp by both sigmoidoscopy and colonoscopy were included. Resection was performed by senior endoscopists with at least five years of experience. If not described in the endoscopy report, polyp morphology was determined by post hoc review of endoscopic images. Only patients with flat polyps (Paris classification 0–Is, 0–IIa, 0–IIb, 0–IIc) were included, those with pedunculated polyps were excluded. For patients who underwent consecutive EMRs of several large polyps, only the first resected polyp was included in the analysis. By applying these criteria, we obtained a list of 802 large polyps from 802 unique patients.

Localization of large colonic polyps was extracted from the endoscopy report. The area proximal of the splenic flexure was defined as the proximal colon and the descending colon, sigmoid and rectum were defined as the distal colon. Histopathology reports provided by the central pathology department were reviewed for histological subtype and grade of dysplasia. Histological findings were assigned into major groups according to Vienna Classification [23]: adenoma with low-grade dysplasia (LGD), adenoma with high-grade dysplasia (HGD) and adenocarcinoma (both invasive and non-invasive). Serrated polyps include hyperplastic polyps, traditional serrated adenomas and/or sessile serrated adenomas/polyps [5, 24]. If a large polyp contained more than one histological subtype, the subtype with the highest degree of dysplasia was chosen. In rare cases, histological findings were corrected after patients underwent surgical removal of the polyp following EMR.

To determine the prevalence and characteristics of synchronous polyps in patients with large polyps, we included only patients who underwent a complete colonoscopy during endoscopic removal of the large polyp. A colonoscopy was considered as complete when an intubation of the caecum was described in the report. If the latter was not described or significant stool contamination was observed, the colonoscopy was considered as incomplete and thus not included. In some cases, a stepwise removal of multiple synchronous polyps in consecutive endoscopic procedures was described. In order to obtain a comprehensive data set on detected synchronous polyps, we collected available reports of colonoscopies performed up to 6 weeks prior and 6 months after EMR of the large polyp. The total number of detected synchronous polyps and their characteristics were then summarized for each patient.

### Statistical analysis

Continuous variables were summarized using mean ± standard deviation. Frequencies (%) were used for categorical variables. Non-parametric Mann–Whitney-Wilcoxon/Kruskal-Wallis test were used to compare continuous parameters between two/multiple groups. Jonckheere-Terpstra test was used to test for trends across groups. Odds ratios including 95 % confidence intervals from logistic regression were used to assess the impact of factors on prevalence of adenoma or synchronous polyps with HGD or adenocarcinoma. Fisher's exact test was used to assess independence between categorical parameters. All tests were two-sided, *p*-values below 0.05 were considered statistically significant. All analyses were performed by using R v3.1 [25].

## Results

### Characteristics of patients and large flat polyps

**Fig. 1:**
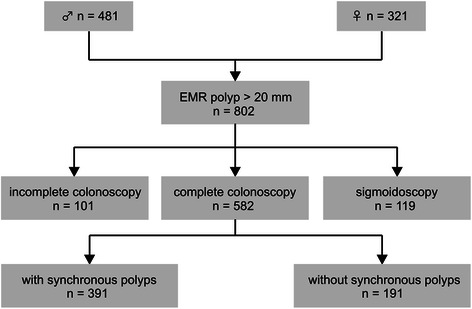
Flow chart showing basic distribution of patients and endoscopic procedures

A total of 802 patients (481 men and 321 women, mean age 65.4 ± 10.5) with 802 colonic polyps >20 mm were included in the analysis. Large flat polyps were removed in 582 cases by complete colonoscopy, in 119 cases by incomplete colonoscopy and in 101 cases by sigmoidoscopy (Fig. 1). Mean size of all large polyps was 34.1 mm (range 20–150 mm, standard deviation 16.1 mm). For all removed large polyps, tissue histopathology was retrieved. The most frequent histological subtypes were adenoma with low-grade intraepithelial dysplasia (LGD) with *n* = 421 (52.5 %), followed by high-grade intraepithelial dysplasia (HGD) with *n* = 214 (26.7 %), adenocarcinoma (both invasive and carcinoma in situ) with *n* = 90 (11.2 %) and serrated polyps (SP) with *n* = 77 (9.6 %). The majority of serrated polyps were hyperplastic polyps or sessile serrated adenomas/polyps with no or low grade dysplasia. Non-serrated adenomas included 254 tubular, 372 tubulovillous and 9 villous adenoma. Large flat polyps were localized in 60.7 % (*n* = 487) in the proximal colon and in 39.3 % (*n* = 315) in the distal colon (see Table 1). Adenocarcinomas in large flat polyps were predominantly stage T1 cancer (Additional file 1: Table S1).

**Table 1:** Baseline characteristics of patients and large flat polyps

Characteristic	Number of patients
Number of patients	802
Age, mean ± SD (range), y	65.4 ± 10.5 (31–92)
Female, no. (%)	321 (40)
Polyp size, mean ± SD (range), mm	34.1 ± 16.1 (20–150)
Polyp location, no. (% of total)	
Caecum	187 (23.3)
Ascending colon	150 (18.7)
Hepatic flexure	71 (8.9)
Transverse colon	79 (9.9)
Splenic flexure	13 (1.6)
Descending colon	40 (5.0)
Sigmoid colon	82 (10.2)
Rectum	180 (22.4)
Histology, no. (% of total)	
Tubular adenoma	254 (31.7)
Tubulovillous adenoma	372 (46.4)
Villous adenoma	9 (1.1)
Adenocarcinoma	90 (11.2)
LGD (without SP with LGD)	421 (52.5)
HGD (without SP with HGD)	214 (26.7)
Serrated polyps	77 (9.6)
Hyperplastic polyps	32 (4)
Sessile serrated adenoma/polyp (SSA/P) without dysplasia	32 (4)
Sessile serrated adenoma/polyp (SSA/P) with LGD	11 (1.37)
Sessile serrated adenoma/polyp (SSA/P) with HGD	2 (0.2)

### Impact of endoscopic and demographic features on histology of large polyps

In order to identify factors that are associated with the occurrence of specific histological subtypes, we analyzed the following parameters: localization and size of flat polyp, patient age and sex. We found no difference in distribution of histological subtypes (*p* = 0.97) between different sex (Table 2). Large polyps containing SP were in average smaller than polyps with other histological subtypes (*p* < 0.0001). In addition, there was a trend towards HGD/adenocarcinoma with increase of polyp size in non-serrated adenomas (significant JT-test, see Table 2). The mean age of patients with SP was lower compared to patients with other histology (59.4 vs 66.1, *p* < 0.0001). We observed a distinct anatomic distribution for polyps with specific histological subtypes. Serrated polyps were predominantly found in the proximal colon (80.5 % of all SP) while adenocarcinomas were preferentially localized in the distal colon (72.2 % of all adenocarcinomas). For LGD or HGD, there was no preference for a specific location within the colon. However, villous adenomas were mainly detected in the distal colon (67 % of all villous adenomas) and tubular adenomas in the proximal colon (74 % of all tubular adenomas) (Additional file 2: Table S2). Localization of polyps also influenced mean size of polyps, with the highest average polyp size in the rectum compared to other sites (44.2 mm vs. 31.2 mm, *p* < 0.0001).

**Table 2:** Characteristics of large flat polyps for histological subtypes

	Histology (total = 802)				
Variable	SP	LGD	HGD	adenocarcinoma	*p* value
Patients, no. (% of total)	77 (9.6)	421 (52.5)	214 (26.7)	90 (11.2)	
Female, no. (% of group with specific histology)	29 (37.7)	171 (40.6)	86 (40.2)	35 (38.9)	0.97
Age, mean ± SD (range), y	59.4 ± 11 (41–87)	65.6 ± 10.3 (31–92)	67 ± 9.6 (38–92)	65.7 ± 11.6 (41–91)	<0.0001
Polyp size, mean ± SD (range), mm	25.6 ± 8.4 (20–70)	31.4 ± 13.8 (20–150)	38.9 ± 15.9 (20–110)	42.5 ± 23.3 (20–126)	<0.0001
Location					
Proximal colon, no. (% of group with specific histology)	62 (80.5)	286 (67.9)	114 (53.3)	25 (27.8)	<0.0001
Distal colon, no. (% of group with specific histology)	15 (19.5)	135 (32.1)	100 (46.7)	65 (72.2)	

### General characteristics of synchronous polyps

Data from complete colonoscopies were available from 582 patients, allowing for a characterization of synchronous polyps. Synchronous polyps were detected in 391 patients undergoing complete colonoscopies (67.2 %). Of those, histological assessment was available in 355 and location described in 378 cases. A total of 1487 synchronous polyps were removed. The mean number of polyps of patients with synchronous polyps was 3.8 (range 1–40). We observed a sex specific difference in the number of detected synchronous polyps. The mean number of polyps was slightly, but significantly higher for male than female (mean 2.0 vs. 2.9, *p* = 0.0029). The total proportion of male patients with at least one synchronous polyp was also higher (71 % vs 61 %, *p* = 0.01). The majority of patients were found to have synchronous polyps at multiple sites within the colon (222 of 378 cases) and there was a general trend toward occurrence in the proximal colon (see Table 3). Interestingly, patients with any synchronous polyps in the rectosigmoid colon had a higher overall polyp burden compared to those with synchronous polyps elsewhere in the colon (mean 3.2 vs. 4.6, *p* < 0.0001). In the majority of cases (243 of 355 colonoscopies), removed synchronous polyps consisted of multiple histological subtypes. Adenocarcinoma was found in 6 %, HGD in 13.8 %, LGD in 85.4 % and SP in 28.2 % of all patients with histologically assessed synchronous polyps (Table 3). The histology of synchronous polyps was associated with overall polyp load, as patients with HGD in any synchronous polyp had a higher average number of polyps (SP: 2.5 vs. HGD: 6.8, *p* < 0.0001).

**Table 3:** Basic characteristics of patients with synchronous polyps and synchronous polyps

Characteristic	Number of patients
Complete colonoscopy, no. patients (%)	582 (100)
with detection of synchronous polyps	391 (67.2)
with location of synchronous polyps	378 (64.9)
with histology of synchronous polyps	355 (61.0)
Synchronous polyp location (% of total)	
Caecum	130 (22.3)
Ascending colon	132 (22.7)
Hepatic flexure	62 (10.7)
Transverse colon	130 (22.3)
Splenic flexure	12 (2)
Descending colon	82 (14.1)
Sigmoid colon	128 (22)
Rectum	83 (14.3)
Synchronous polyp histology (% of total)	
Serrated polyps (including hyperplastic polyps)	100 (17.2)
LGD	303 (52.1)
HGD	49 (8.4)
Adenocarcinoma	21 (3.6)

### Factors associated with occurrence of high-grade dysplasia and adenocarcinoma

Based on our data, we sought to identify factors that were associated with the occurrence of adenoma with HGD/adenocarcinoma, for both large flat polyps and synchronous polyps. By multivariate logistic regression analysis based on all patients with synchronous polyps, we found that increase in polyp size (OR 1.29, 95 % CI 1.09–1.55, per 10 mm increase, *p* = 0.0041), location of the large polyp in the rectosigmoid colon (OR 3.89, 95 % CI 2.26–6.79, *p* < 0.0001) and increase in age (OR 1.13, 95 % CI 1.00–1.29, per 5 year increase, *p* = 0.0471) were independently associated with presence of HGD/adenocarcinoma in large polyps. In contrast, patient sex, the location and the number of synchronous polyps had no significant effect on histology of the large polyp (Table 4). Biopsies of polyps prior to EMR did not detect the presence of adenocarcinoma in most cases (see Additional file 1: Table S1).

**Table 4:** Logistic regression models for occurrence of HGD/adenocarcinoma in flat polyps >20 mm

Multivariate logistic model			
Parameter	N	OR (lower – upper 95 % CI)	*p*-value
Increase in size of large polyp (per 10 mm)	355	1.29 (1.09–1.55)	0.0041
Increase in age (per 5 years)		1.13 (1.00–1.29)	0.0471
Female vs. male sex		1.24 (0.74–2.07)	0.41
Location in rectosigmoid colon vs. other location in the colon		3.89 (2.26–6.79)	<0.0001
Increase in number of synchronous polyps (per 1 polyp)		0.95 (0.88–1.01)	0.10
Any synchronous polyp with HGD or adenocarcinoma vs. other histology		3.21 (1.73–6.06)	0.0003

We also analyzed parameters potentially associated with occurrence of HGD/adenocarcinoma in synchronous polyps (Table 5). Location of any synchronous polyp in the rectosigmoid colon (OR 2.65, 95 % CI 1.44–5.0, *p* = 0.002) and a high number of synchronous polyps (OR 1.16, 95 % CI 1.09–1.24, *p* < 0.001) were independently associated with HGD/adenocarcinoma. Interestingly, presence of HGD/adenocarcinoma in the large polyp (OR 3.33, 95 % CI 1.77–6.35, *p* = 0.0002) was also associated with occurrence of HGD/adenocarcinoma in synchronous polyps. In contrast, location of the large polyp in the rectosigmoid colon was not associated with the occurrence of HGD/adenocarcinoma in our multivariate analysis.

**Table 5:** Logistic regression models for occurrence of HGD/adenocarcinoma in any synchronous polyp

Multivariate logistic model			
Parameter	n	OR (lower – upper 95 % CI)	*p*-value
Increase in size of large polyp (per 10 mm)	352	1.14 (0.94–1.38)	0.17
Increase in age (per 5 years)		0.97 (0.84–1.12)	0.66
Female vs. male sex		1.01 (0.53–1.86)	0.98
Increase in number of synchronous polyps (per 1 polyp)		1.16 (1.09–1.24)	<0.0001
large polyp with HGD or adenocarcinoma vs. other histology		3.33 (1.77–6.35)	0.0002
Location of large polyp in rectosigmoid colon vs. other location in the colon		0.84 (0.40–1.67)	0.62
Location of any synchronous polyp in the rectosigmoid colon vs. other location		2.65 (1.44–5.0)	0.002

## Discussion

### Characteristics of large flat colorectal polyps

By retrospectively investigating a large single center cohort, we show that flat colonic polyps >20 mm have a distinct distribution of histology and localization. Overall, our results demonstrate that large flat colonic polyps preferentially occur in the proximal colon, as shown by others [11, 13, 16]. Large flat colonic polyps containing invasive and non-invasive adenocarcinoma are found at a frequency of 2.5–7 % [11, 12, 15, 17]. The percentage of adenocarcinoma in our cohort was slightly higher (11.2 %), possibly due to the high fraction of rectal polyps, which we found to contain adenocarcinoma more frequently. The percentage of polyps containing adenoma with high-grade dysplasia varied greatly between different studies, ranging from 7.5 % to 40.5 % [14, 15, 17]. This divergence among studies may reflect discrepancies in selection criteria of polyps. For example, some studies excluded rectal polyps [16] while others did not [14]. The fraction of HGD in our cohort (26.7 %) was within the reported range. The rate of sessile serrated adenomas/polyps was shown to be less than 5 % in patients undergoing screening colonoscopy [21, 26]. We found a higher rate of serrated polyps in our cohort (9.6 %). This may reflect the true prevalence of SP within large colonic polyps, which is supported by a recent study that also found a higher frequency of 20 % [12]. The correlation between histology and location of large colonic polyps showed that large adenomas with HGD and adenocarcinomas are predominantly located in the rectosigmoid colon, which is in line with previous observations [11, 13, 16]. While small hyperplastic polyps are mostly left sided, sessile serrated adenomas/polyps are located predominantly in the proximal colon [5]. Indeed, the majority of SP in our cohort were sessile serrated adenomas/polyps and we found a preference towards a right-sided location. We identified polyp size and age to be independently associated with advanced dysplasia and carcinoma in large polyps. Polyp size has been known to correlate with malignant conversion, with rates of HGD increasing significantly for polyps >20 mm [7, 27]. Advanced patient age has also been previously described to be associated with HGD in adenomatous polyps [10]. Our data shows that patients with large flat, serrated polyps were significantly younger than patients with large polyps of other histology. The majority of SP in our cohort harbor no dysplasia or only low grade dysplasia and this subgroup of SP is known to preferentially occur in younger patients [26]. In summary, our cohort of patients represents clinicopathological features that support previous observations for large flat adenomas.

### Prevalence and characteristics of synchronous polyps

A main focus of this study was to investigate how specific features of large polyps affect prevalence and characteristics of synchronous polyps. Synchronous polyps are reported to occur in 21–29 % of patients undergoing screening colonoscopy [28, 29] and 36 % with colorectal cancer [19]. The rate of synchronous polyps was much higher in our cohort (67.4 %), indicating that large polyps are generally associated with synchronous polyps. A potential link between the histology of large polyps and synchronous polyps was proposed by reports showing that large proximal SP were associated with synchronous advanced dysplasia [20, 22]. Our study demonstrates that presence of HGD in the large polyp is strongly associated with co-occurrence of synchronous polyps with the same histology. This observation is supported by a study showing that occurrence of adenomas with advanced dysplasia in the distal colon is predictive of advanced lesions in total colonoscopy [30]. In addition, we found that HGD in any synchronous polyp was also associated with an increased overall number of synchronous polyps. These observations provide further clinical evidence for the theory of field carcinogenesis, which implies that the environmental milieu that leads to carcinogenesis is not a local event, but affects larger parts of the colon [31].

### Limitations of the study

The majority of large flat polyps in our cohort was detected by primary or secondary (gastroenterological) centers and then referred to our institution for removal. This might introduce a selection bias towards polyps that are most difficult to resect, due to their morphology or location, e.g., in the proximal colon. In some cases, there was documented removal of small, synchronous polyps by external gastroenterologists prior to admission. We must therefore assume that the fraction of patients with synchronous polyps and the number of polyps per patients are underestimated by our study. In addition, a considerable proportion of colonoscopies could not be included in our analysis of synchronous polyps, as they were considered incomplete. This may introduce a selection bias, but comparison of clinic-pathological characteristics of polyps between the complete and incomplete colonoscopy group did not reveal significant differences (data not shown). Due to the retrospective design, there was no predefined scheme for the documentation of polyp morphology. In some cases, polyp morphology was determined by post hoc review of endoscopic images which is prone to inter-observer variation and thus imprecise. Furthermore, quality of bowel preparation and polyp detection rate of endoscopists were not documented in a standardized manner. Therefore, these parameters could not be comprehensively assessed in our retrospective approach. In addition, this study includes data from a period of ten years. During this time, improvements to endoscopy imaging technologies have been introduced which partially increased adenoma detection rates [32, 33]. In summary, these limitations indicate that the rate of synchronous polyps is likely underestimated due to our retrospective approach.

### Clinical impact

What is the impact of this finding for clinical practice? First, our data underlines the importance of a complete colonoscopy prior to removal of large polyps, especially if high grade dysplasia or malignancy is highly suspected due to the presence of “red flags” (advanced age, localization in the rectosigmoid, large size). Secondly, surveillance after resection should always include a complete colonoscopy and not be restricted to the site of the large polyp. This is of particular importance as the incidence of metachronous cancer is higher in patients with synchronous polyps [34–36]. Lastly, our data supports that endoscopic instead of surgical removal of large polyps should be considered first. Surgical removal of large polyps is associated with a considerable level of post-operative complications [37] and may not be ideal to remove synchronous or metachronous adenomas at multiple sites. In contrast, endoscopic resection allows for repetitive and multilocal removal. If endoscopic removal fails, surgery can still be performed without compromising oncological outcome [38].

## Conclusions

In summary, our retrospective analysis shows that the prevalence of synchronous polyps is high in patients with large polyps and that occurrence of large polyps with HGD/adenocarcinoma significantly correlates with presence of synchronous polyps containing high-grade dysplasia and adenocarcinoma. These findings underline the importance of a complete colonoscopy prior to endoscopic removal and during follow-up of patients with large colonic polyps.

## Additional files

Additional file 1: Table S1. Diagnostics prior to EMR and local staging of large flat colonic polyps with adenocarcinoma. Additional file 2: Table S2. Characteristics of large flat colonic polyps with tubular, tubulovillous and villous adenoma.

## Abbreviations

EMR: Endoscopic mucosal resection
LGD: (adenoma with) Low-grade dysplasia
HGD: (adenoma with) High-grade dysplasia
SP: Serrated polyps
OR: Odds ratio
CI: Confidence interval
aCA: Adenocarcinoma
