# Supplementary material for: Frequent co-occurrence of high-grade dysplasia in large flat colonic polyps (>20 mm) and synchronous polyps
Source: BMC Gastroenterol. 2015 Jul 10;15:82. doi: 10.1186/s12876-015-0312-4 (PMC4498525; doi:10.1186/s12876-015-0312-4)
Supplement: Additional file 2: Table S2. — Characteristics of large flat colonic polyps with tubular, tubulovillous and villous adenoma. [file 12876_2015_312_MOESM2_ESM.docx]

**Supplementary Table 2: Characteristics of large flat colonic polyps with tubular, tubulovillous and villous adenoma.**

|  | **Tubular**  **adenoma** | **Tubulovillous adenoma** | **Villous**  **adenoma** |
| --- | --- | --- | --- |
| Patient, no. | 254 | 372 | 9 |
| Age, mean ± SD (range), y | 66.3 ± 9.9 | 66.1 ± 10.2 | 61.4 ± 13.2 |
| Female, no. (%) | 99 (39) | 154 (41) | 4 (44) |
| Polyp size, mean ± SD (range), mm | 29.4 ± 10.1 | 36.9 ± 16.9 | 41.1 ± 15.4 |
| ***Histology*** |  |  |  |
| LGD, no. (% of group with specific histology) | 191 (75) | 223 (60) | 7 (78) |
| HGD, no. (% of group with specific histology) | 63 (25) | 149 (40) | 2 (22) |
| ***Location*** |  |  |  |
| Proximal colon, no. (% of group with indicated histology) | 188 (74) | 209 (56) | 3 (33) |
| Distal colon, no. (% of group with indicated histology) | 66 (26) | 163 (44) | 6 (67) |
| ***Synchronous polyps*** |  |  |  |
| patients with synchronous polyps, no. (% of patients with complete colonoscopies and indicated histology) | 143 of 205 (70) | 165 of 257 (64) | 5 of 7 (71) |
| synchronous polyps, mean no., ± SD (range); only complete colonoscopies included | 2.87 ± 3.87 (0-26) | 2.5 ± 4.47 (0-40) | 3.29 ± 4.3 (0-13) |
